# Antirepressor specificity is shaped by highly efficient dimerization of the staphylococcal pathogenicity island regulating repressors: Stl repressor dimerization perturbed by dUTPases

**DOI:** 10.1038/s41598-024-51260-y

**Published:** 2024-01-23

**Authors:** Kinga Nyíri, Enikő Gál, Máté Laczkovich, Beáta G. Vértessy

**Affiliations:** 1 Department of Applied Biotechnology and Food Science, Faculty of Chemical Technology and Biotechnology, Budapest University of Technology and Economics, Műegyetem rkp. 3., Budapest, 1111 Hungary; 2 Institute of Molecular Life Sciences, HUN-REN, Research Centre for Natural Sciences, Magyar Tudósok Krt 2., Budapest, 1117 Hungary

**Keywords:** DNA-binding proteins, Viral proteins

## Abstract

The excision and replication, thus the life cycle of pathogenicity islands in staphylococci are regulated by Stl master repressors that form strong dimers. It has been recently shown that SaPIbov1-Stl dimers are separated during the activation of the *Staphylococcus aureus* pathogenicity island (SaPI) transcription via helper phage proteins. To understand the mechanism of this regulation, a quantitative analysis of the dimerization characteristics is required. Due to the highly efficient dimerization process, such an analysis has to involve specific solutions that permit relevant experiments to be performed. In the present work, we focused on two staphylococcal Stls associated with high biomedical interest, namely Stl proteins of *Staphylococcus aureus* bov1 and *Staphylococcus hominis* ShoCI794_SEPI pathogenicity islands. Exploiting the interactions of these two Stl proteins with their antirepressor-mimicking interaction partners allowed precise determination of the Stl dimerization constant in the subnanomolar range.

## Introduction

Repressors are molecular switches, which typically function through binding to specific DNA segments thus directly blocking transcription at a specific site to suppress the expression of the encoded protein(s). Bacteria apply repressors to adapt to environmental changes for example to fine tune their metabolism based on the available nutrients. Temperate phages, prophages and other mobile genetic elements such as pathogenicity islands also employ repressors to regulate their life cycle. While the most common mechanism to relieve the bacterial repressors involves small molecules that bind to the repressor protein resulting in conformational change that changes their DNA binding affinity, phage repressors are usually relieved via proteolytic cleavage of the protein^1^. This process also constitutes an environmental adaptation since cleavage is activated by the bacterial SOS response caused by massive DNA damage. In such cases, the phage progresses from lysogenic growth to lytic growth to maintain its genomic integrity.

Interestingly, unlike most temperate phage repressors, the main repressors of *Staphylococcus aureus* pathogenicity islands (SaPIs), although considered as virus satellites^2^, are not activated directly by SOS response. The derepression of these SaPI repressors is exerted through direct interaction of the master repressor protein, termed Stl, with a specific helper phage protein^2^. Similarly, some P2 related phages are also relieved via a complex formation with an antirepressor protein^3–5^.

Despite their differences in activation, oligomerization is a common characteristic of bacterial, phage and SaPI repressors alike, allowing for more sensitive and complex regulation mechanisms^6,7^.To exert their function, repressor proteins typically contain a DNA-binding domain (on the N-terminal of the protein), which recognizes specific DNA sequences, and a multimerization domain (on the C-terminal of the protein), which mediates the formation of the repressor dimers or higher order oligomers. The dissociation equilibrium constant of the repressor oligomer formation falls within a wide range between μM to pM^8–11^.

If derepression is not caused by proteolysis, then antirepressors function by either (i) directly disturbing the DNA binding segment of the repressor protein like in the case of the Sri antirepressor protein of the 80α phage and the Stl repressor of SaPI1 *Staphylococcus aureus* pathogenicity island^12^ or (ii) perturbing the oligomerization of the repressor as in the case of several phage dUTPases and SaPIbov1 Stl (Sa-Stl)^2,13–15^. In such cases the antirepressor-repressor interaction has to be stronger than the protein–protein interactions on the oligomer interface. It is important to recognize that in such systems, two types of complexation has to be characterized simultaneously: on the one hand, the antirepressor-repressor complexation and on the other hand, the repressor oligomerization. Hence, there is an inherent, and rarely addressed, difficulty in the quantitative analysis of these gene expression regulation mechanisms. Namely, in the lack of specific knowledge of the dimerization (oligomerization) dissociation constant, it is hard to describe in detail the interaction between the antirepressor and the repressor. Therefore, determination of the dimerization equilibrium is of crucial importance. In case of high affinity oligomerization, it is not a straightforward task and needs tailored solutions. It has been shown that 0.3–40 nM affinity of Sa-Stl can be measured to its antirepressor dUTPases bound to a sensor surface^15–17^. Within these assays Sa-Stl dimerization was not considered thus the strength of interaction is underestimated.

In this work, we set out to quantitatively characterize dimerization of two different SaPI Stl proteins using “antirepressor-mimicking” human and mycobacterial dUTPases as in vitro tools. SaPI segments are frequently involved in spreading toxins and virulence factors within and beyond staphylococci. Studying the mechanism of their regulation by Stl proteins is therefore of great biomedical significance.

## Methods

### Cloning and mutagenesis

The gene encoding Stl repressor of *Staphylococcus hominis* ShoCI794_SEPI pathogenicity island (Sho-Stl, WP_049379208) was synthesized by Gene Universal and delivered in pGEX-4T-1 vector in frame with an N-terminal GST and a thrombin site. The K99N, E100D, F105Y mutations were introduced to Sho-Stl in two steps by using NEB Q5 mutagenesis kit to result in the triple mutant protein Sho-Stl-NDY, which has the same dUTPase binding motif as Sa-Stl.

Enzymatically biotinylated dUTPase and Stl proteins were created by introducing a so-called Avi-tag (MSGLNDIFEAQKIEWHE) to the N- and C-terminus of the proteins, respectively. The genes encoding the human and mycobacterial dUTPases were cloned into a PAN4 vector in frame with the N-terminal Avi-tag. NEB Q5 insertion mutagenesis protocol was used to insert the Avi-tag to the C-terminus of both Stl proteins. The sequence of every construct was verified by sequencing.

### Protein expression and purification

The Sa-Stl, the human and mycobacterial dUTPase proteins were expressed and purified as described in our previous work^13,18^. To gain biotinylated human and mycobacterial dUTPases, the proteins with an Avi-tag implemented at its N-terminus were expressed in *E. coli* AVB101 cells in 500 ml LB supplemented with 100 µg/ml ampicillin and 10 µg/ml chloramphenicol, at OD_600_ = 0.6 we added 1.5 mM IPTG and expressed the protein at 18 °C overnight. Biotinylation of the dUTPases was achieved during expression by adding 50 µM biotin to the culture, the protein was then purified by Ni^2+^-affinity chromatography^13^. Avi-tagged Sa-Stl and Sho-Stl proteins were expressed and purified as described by Nyiri *et al*^13^ and Hirmondó *et al*^18^ during the purification 50 µM biotin and a pellet of E*. coli* AVB101 cultured overnight at 18 °C was added to the pellet of Stl to include the biotinylating enzyme (BirA) in the lysate.

### Steady state dUTPase activity assay

The steady state dUTPase enzyme activity in the presence and absence of 300 nM of Sa-Stl or Sho-Stl was measured by following the absorbance change of phenol red at 559 nM due to the proton release during the dUTP hydrolysis in the assay buffer (1 mM HEPES, 150 mM KCl, 5 mM MgCl_2_, 40 µM phenol red, pH = 7.5). Initial velocity was determined from the slope of the first 10% of the progress curve.

### Biolayer interferometry

The Stl:dUTPase binding was characterized by an Octet K2 system (Sartorius), using streptavidine coated SAX biosensors (Sartorius). Experiments were performed at 30 °C using 4titude, Black Solid Bottom Assay Plates and shaker speed of 1000 rpm. The association and dissociation of the analyte protein (Stl or dUTPase) was followed in a buffer containing 50 mM HEPES, 300 mM NaCl, 5 mM MgCl_2_, pH 7.5 applying at least five different dilutions. Data analysis was carried out with Octet Data Analysis HT program (Sartorius) applying a 1:1 binding model. Curves and fitting is displayed on Figs. S2–S8 in the Supporting information.

### Protein modelling

The structural model for the dimer of C-terminal segment of ShoCI794-Stl (residues 164–256) was generated with AlphaFold2 default mode using ColabFold v1.5.1^19^. Dimerization surface was studied by LigPlot + and PDBePISA^20,21^.

## Results and discussion

### Binding kinetics of SaPIbov1 Stl to human dUTPase reveals details on Stl dimerization

It has been shown that the apparent K_i_ of Stl protein of SaPIbov1 pathogenicity island** (**Sa-Stl) on human dUTPase is 6.7 ± 2.7 nM^13^. Thus to measure the binding kinetics of the complex with biolayer interferometry an even stronger interaction (with subnanomolar K_D_) is needed to fix one of the partners to the sensor to avoid the release of the whole protein–protein complex from the sensor in the dissociation step, which would lead to overestimation of the dissociation rate and underestimation of dissociation equilibrium constant. The equilibrium dissociation constant of His_6_-tag to Ni–NTA surface was found to be 14 nM, which is in the range of the measured apparent K_i_^22^. Therefore we applied enzymatically biotinylated human dUTPase and Stl proteins and high precision streptavidin biosensors (SAX, Sartorius) in the BLI assays, since the biotin–streptavidin interaction is one of the strongest non-covalent interactions (K_D_ = 0.04 pM)^23^.

We determined the equilibrium dissociation constant of (K_D_) of the Sa-Stl and human dUTPase complex to be 9.75 ± 0.04 nM (k_a_ = 6.67 ± 0.03·10^4^ M^−1^ s^−1^, k_dis_ = 6.50 ± 0.01·10^–4^ s^−1^) with biolayer interferometry, by using specifically biotinylated human dUTPase as a sensor (biotin was attached to the N-terminus of the protein, which is not interacting with Sa-Stl^13^, previous studies used N-terminal His-tag for binding of dUTPase to the sensor^15,16^). This was in good agreement with the K_i_ measured in our previous study^13^. We decided to specifically biotinylate the C-terminus of Sa-Stl which is not involved in the human dUTPase-Stl complex surface according to our HDX-MS results^13^. When biotinylated Sa-Stl was bound to the sensor and the binding kinetics of human dUTPase was examined we got K_D_ = 0.18 nM.

(k_a_ = 1.53 ± 0.003·10^6^ M^−1^ s^−1^, k_dis_ = 2.73 ± 0.02·10^−4^ s^−1^).

This dramatic difference between the two observed affinities can be explained if we consider the role of Sa-Stl dimerization in dUTPase binding. It has been shown that Sa-Stl binds to dUTPases as a monomer, while it forms dimers otherwise^12–15^.

**Figure 1:**
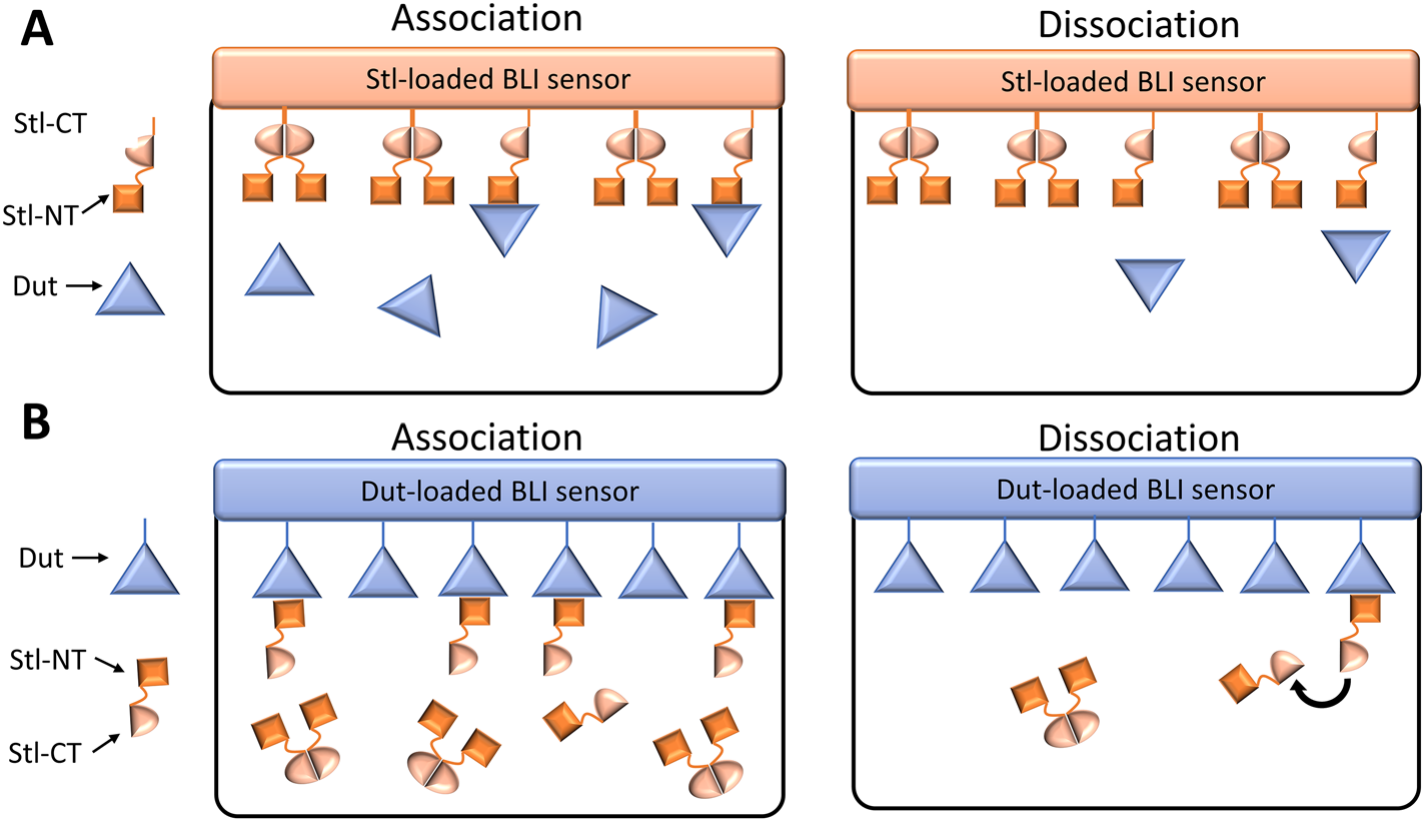
Schematic representation of biolayer interferometry experiments. (**A**) Stl protein biotinylated on its C-terminus and bound to the sensor. The unbiased association and dissociation of human dUTPase is followed during the experiment. (**B**) Human dUTPase protein biotinylated on its N-terminus and bound to the sensor. Association and dissociation of Stl is perturbed by Stl dimerization.

When Stl is bound to the sensor the dimerization does not perturb the binding as only the monomers interact with the dUTPase and the dimers are inert (Fig. 1A). On the other hand if dUTPase is bound to the sensor (Fig. 1B): (i) the effective Stl concentration is much lower than the nominal Stl concentration as only monomers of Stl can bind dUTPase (thus k_a_ is smaller, than in the reverse setup, as here it is calculated with the nominal Stl concentration) and (ii) the dimerization equilibrium facilitates the dissociation of Stl from the sensor (k_dis_ is larger, than in the reverse setup). The dimerization equilibrium constant of Sa-Stl can be estimated by considering the difference in the association rate of this two BLI measurements. The two k_a_ values should be equal, if the correct actual Stl monomer concentration is included during the evaluation of Sa-Stl binding curves to human dUTPase loaded sensors. Probing different dimerization equilibrium constants to calculate the monomer Stl concentration, it turned out that if K_DIM_ of ca. 0.12 nM was applied the two k_a_ values were close to equal. This is consistent with our previous gelfiltration, SAXS, equilibrium AUC experiments, in which we were not able to detect Stl monomers due to strong dimerization.

With this method we could determine the dimerization dissociation constant of the SaPIbov1 Stl protein as 0.12 nM. It is also of interest to see if other Stls also share similar strong dimerization characteristics. Stls of different SaPIs share only limited sequence identity (7–37%)^24^, however due to horizontal transfer of SaPIs to other species, Stl proteins of 6 other pathogenicity islands show 41–99% identity to Sa-Stl^25^ Out of these Stl homologues we selected to study the Stl of ShoCI794_SEPI (later Sho-Stl) of *Staphylococcus hominis*, that is known to interact with dUTPases, to see if it has similar dimerization characteristic to that of Sa-Stl.

### Stl proteins of *S. aureus* SaPIbov1 and *S. hominis* ShoCI794_SEPI pathogenicity islands differ in dUTPase binding but similar in dimerization properties

**Figure 2:**
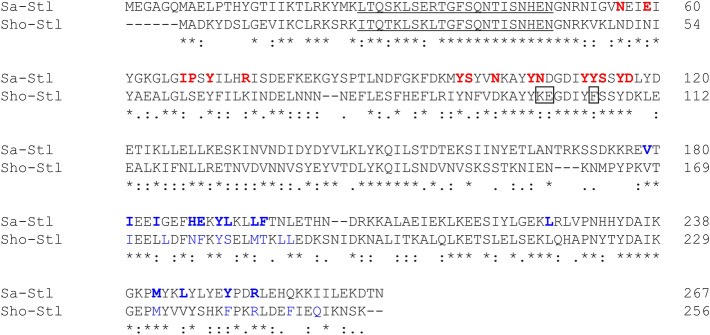
Sequence alignment of Stl master repressor proteins of *S. aureus* SaPIbov1 (Sa-Stl) and *S. hominis* ShoCI794_SEPI (Sho-Stl) pathogenicity islands. Identical residues are marked with a star (*), strongly similar ones denoted with a colon (:) weakly similar residues marked with a dot (.). Residues constituting the DNA binding helix-turn-helix motif are underlined. Sa-Stl residues interacting the human dUTPase based on crystal structure of the Sa-Stl:human dUTPase complex (PDB ID: 7PWJ) are with bold red letters. Residues on the dimer interface of Sa-Stl (PDB ID: 6H48) are with bold blue letters and those of Sho-Stl (modelled in this study by Alphafold2) interface identified are colored blue (both based on by LigPlot + analysis). Sho-Stl residues which were mutated in this study (K99N, E100D, F105Y) are framed.

The sequence of Stl proteins of *S. aureus* SaPIbov1 and *S. hominis* ShoCI794_SEPI pathogenicity islands has 42% sequence identity, while 25% and 9% of their residues are strongly and weakly similar respectively (Fig. 2). We verified by chemical crosslinking and gelfiltration that Sho-Stl forms dimers in solution (Fig. S1 in the Supporting information).

We also studied the effect of Sho-Stl on the enzymatic activity of human dUTPase, and it turned out that Sho-Stl does not inhibit the human dUTPase. This could happen either because of a weaker dUTPase:Stl interaction or due to the strong dimerization of Sho-Stl or the combination of these two effects.

To access the dimerization equilibrium constant of Sho-Stl we performed BLI measurements with the human dUTPase and Sho-Stl. When C-terminally biotinylated Sho-Stl was bound to the sensor and the association of human dUTPase was followed the K_D_ appeared to be 4.5 nM (Table 1, k_a_ = 5.2·10^4^ M^−1^ s^−1^, k_dis_ = 2.5·10^–4^ s^−1^). Based on this BLI result the Sho-Stl: human dUTPase interaction is substantially weaker than the binding of the human dUTPase to Sa-Stl (K_D_ = 0.18 nM). To rationalize this difference we compared the Sa-Stl residues interacting the human dUTPase based on crystal structure of the Sa-Stl:human dUTPase complex (PDB ID: 7PWJ) with the corresponding Sho-Stl residues (Fig. 2, Table 2). This analysis revealed that Sho-Stl is incapable to form interaction with the human dUTPase at several positions. We introduced K99N, E100D, F105Y mutations in Sho-Stl to perfectly match with the Y105-D120 region of Sa-Stl, which segment is proved to form interactions with the active site of various dUTPases^13,15,26,27^. This new triple mutant construct, named Sho-Stl-NDY binds to human dUTPase with a K_D_ of 7.6 nM (Table 1). Thus with these changes we were unable to enhance the binding of Sho-Stl to human dUTPase, which suggests that the Stl:dUTPase interaction have several other key components outside of the interaction of the dUTPase active site with Stl.

**Table 1:** Biolayer interferometry data on dUTPase:Stl complexes (Curves and fitting is displayed on Figures S2-S8 in the Supporting information).

sensor	analyte	K_D_ (nM)*	k_a_ (M^−1^ s^−1^)*	k_dis_ (s^−1^)*	χ^2#^
hDUT	Sa-Stl	9.75	6.7·10^4^	6.5·10^–4^	3.07
Sa-Stl	hDUT	0.18	1.5·10^6^	2.7·10^–4^	1.20
Sho-Stl	hDUT	4.54	5.2·10^4^	2.3·10^–4^	0.57
Sho-Stl-NDY	hDUT	7.62	3.1·10^4^	2.4·10^–4^	4.54
hDUT	Sho-Stl	Strongly distorted binding curves			
Sho-Stl	mtDUT	0.013 ± 0.008^§^	2.2·10^4^	2.9·10^–7^ ± 1.8^§^	0.12
mtDUT	Sho-Stl	35	1.2·10^4^	4.1·10^–4^	0.48

hDUT: human dUTPase; Sa-Stl: *S. aureus* SaPIbov1 Stl; Sho-Stl: *S. hominis* ShoCI794_SEPI Stl; Sho-Stl-NDY: K99N, E100D, F105Y triple mutant Sho-Stl, which has the same dUTPase binding motif as Sa-Stl, mtDUT: *Mycobacterium tuberculosis* dUTPase.

*If not shown the error of fitted values was below 1%

^#^χ^2^ < 5 statistics considered as a good fit.

^§^Close to the detection limit.

**Table 2:** Residues of Sho-Stl protein that differ from the corresponding Sa-Stl residues within the Sa-Stl: human dUTPase complex interface based on crystal structure (PDB ID: 7PWJ).

hDUT	Sa-Stl		Sho-Stl	
Residue-atom	Residue-atom	Type of interaction with hDUT	Corresponding residue	Potential interaction with hDUT
I119-Cγ2	E59-Cδ	vdW	N53	**–**
F135-Cβ	I67-Cγ2	vdW	L61	Possible
F135-Cε	P68-Cδ	vdW	S62	Possible
28Y-OH	R74-NH1	H-bond	K68	Possible
133G-C and 134G-Cα	S99- Cβ	vdW	N91	Potential steric clash
D127-Oδ1	N107-Oδ1	H-bond	K99*	Potential steric clash
G131-O	N107-Nδ2	H-bond	K99*	**–**
G87-O	Y113-OH	H-bond	F105*	**–**
G87-N	Y113-OH	H-bond	F105*	**–**

*Residue mutated in Sho-Stl-NDY construct.

When the human dUTPase were fixed on the BLI sensor the association and dissociation of both Sho-Stl and Sho-Stl-NDY proteins resulted in strongly distorted association curves, resembling the curves obtained if there is mass transport limitation in the system. We observed signals only if we applied 1–12 μM concentration of Sho-Stl and Sho-Stl-NDY which is thousand times excess of the K_D_ values measured in case of the reverse setup. We hypothesized that the dimerization of Sho-Stl can strongly limit the amount of the analyte (ie. monomer form of Sho-Stl) in this setup because the human dUTPase Sho-Stl interaction K_D_ is well above the subnanomolar range. Therefore, we tested if we could assess information about the dimerization of Sho-Stl if we study its interaction with the mycobacterial dUTPase, which is a potentially stronger binding partner (Table 1). If Sho-Stl was bound to the sensor and the binding of mycobacterial dUTPase was characterized with a K_D_ of ca. 13 pM, which is close to the detection limit of the method.

Similarly to the case of human dUTPase:Sa-Stl the measured K_D_ for mycobacterial dUTPase:Sho-Stl substantially differ depending on which protein is fixed on the sensor due to Sho-Stl dimerization. When the mycobacterial dUTPase was attached to the sensor the K_D_ of binding of Sho-Stl was found to be 35 nM (Table 1). Comparing the association kinetics of the latter two experiments the dimerization did not have a strong effect on the association rate constants of Sho-Stl to the mycobacterial dUTPase, perhaps due to the slow binding of the complex (k_on_ = 2.2·10^4^ M^−1^ s^−1^) the dimerization did not limit the available Sho-Stl monomers. Analyzing the dissociation kinetics obtained in the two BLI experiments with mycobacterial dUTPase and Sho-Stl, the data clearly indicate that Sho-Stl dimerization facilitates the dissociation of this protein from the mycobacterial dUTPase-loaded sensor (Table 1). Although the large difference in k_off_ values might also refer to some mechanistic differences.

**Figure 3:**
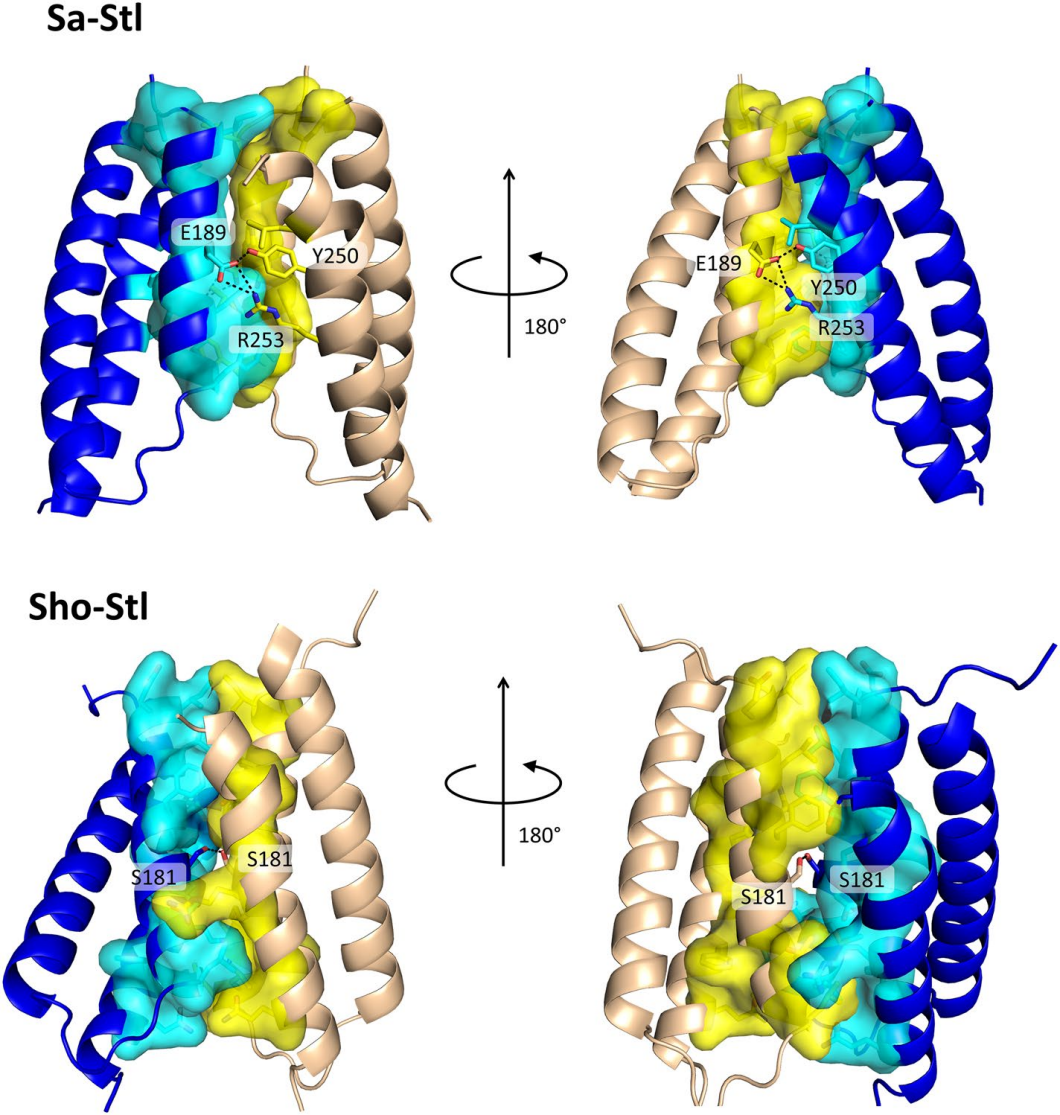
X-ray diffraction and AlphaFold2 models of dimerization segments of Sa-Stl and Sho-Stl, respectively. Proteins are represented as cartoon, residues forming H-bond interactions are labeled and shown as sticks with atomic coloring (C:yellow/cyan, O:red, N:blue, S:orange). Hydrogen bonds are represented with black dotted lines. Residues involved in van der Waals interactions are shown as sticks with atomic coloring (cf. above) and surface, (except for L245 of Sa-Stl, which is shown only as sticks to ease visualization). Regions missing from the crystal structure of Sa-Stl are omitted from the corresponding in silico model of Sho-Stl. Residues shown here are also indicated on Fig. 2. (PDB ID for Sa-Stl dimer: 6H48.)

To assess information about the Sho-Stl dimerization the structure of the Sho-Stl dimer was modeled by AlphaFold2 and the dimer interface of this structural model was compared to that of Sa-Stl based on X-ray crystallography (PDB ID:6H48)^15^. The area of the dimerization surface of Sho-Stl (residues 164–256) and Sa-Stl (residues 175–267) was estimated to be 836 Å^2^ and 773 Å^2^ respectively by PDBePISA^20^. The solvation free energy gain upon formation of the interface was calculated to be 15.5 kcal/mol and 12.9 kcal/mol for Sho-Stl and Sa-Stl. The Sa-Stl dimers are also held together by 3 hydrogen-bonds between residues Glu189 and Tyr250; Glu189 and Arg253 of both chains symmetrically, while in case of Sho-Stl only one such bond was found between the Ser181 residues of the two protomers (Fig. 3). Although the formation of dimers results in less solvation free gain in case of Sa-Stl, than that of Sho-Stl, due to the two additional hydrogen bonds its dimerization free energy could be similar to that of Sho-Stl, as in general H-bond energy of proteins is around 0.5–1.5 kcal/mol^28^. The number and type of hydrophobic residues at the dimer interface is also predicted to be similar by Ligplot + ^21^ (Fig. 3).

A rough estimate of dimerization equilibrium constant of Sho-Stl can be calculated based on the lack of inhibition effect of that on human dUTPase. If we assume that the substrate dUTP (dUTPase:dUTP K_D_ ≤ 1 μM^29^) is a weak competitor of Stl, then the residual dUTPase activity is due to the Stl-free dUTPase active sites. Based on Sho-Stl:human dUTPase K_D_ = 4.5 nM and remaining activity (close to 100%) of 50 nM human dUTPase at 200 nM and 300 nM Sho-Stl concentration the competing dimerization would have to be characterized with a K_DIM_ of 1 pM, which is due to the assumptions likely to be an overestimate. If we apply the same K_DIM_ for Sa-Stl the calculated active fraction of 50 nM human dUTPase at 200 nM and 300 nM Sa-Stl concentration is 37% and 32%, which agrees well with the experimental results^13^.

Considering that the same K_DIM_ value reproduces the results of inhibition assays and the similarity of the dimerization surface of Sho-Stl model to that of Sa-Stl, we suggest that the two proteins have very similar dimerization equilibrium constants, which based on our BLI results should be in a subnanomolar range.

The level of strength of Stl dimerization is matching with that of other repressors, although only few quantitative data on repressor dimerization equilibrium is available so far, possibly due to the technical difficulties of obtaining such information (Table 3). In most cases dimerization equilibrium constants of repressors were acquired from gelfiltration chromatograms of radioactively-labeled proteins. On the other hand, dimerization equilibrium constant of Lac repressor from *E. coli* is deduced from kinetic data of repressor-DNA binding. We also gained information about Stl repressor dimerization indirectly, by studying its interaction with antirepressor-mimicking dUTPases. We suggest that this methodology can be generally used for other repressors.

**Table 3:** Dimerization equilibrium constants of bacterial and phage repressors.

organism	Repressor name	K_D_	Method	Ref.
λ phage	cI	5.6 nM	Radioactive labeling and gelfiltration	^8^
λ phage	Cro	3 μM	Radioactive labeling and gelfiltration	^9^
*E. coli*	LacR	~ 10 pM	Estimate from observed protein-DNA k_a_ at different concentrations	^10^
*S. aureus* phage ϕ11	cI	< 10 nM*	Gelfiltration	^11^
SaPIbov1	Stl	0.12 nM ~ *1 pM*	BLI *BLI and DUT inhibition*^*#*^	In this work
ShoCI794_SEPI	Stl	~ *1 pM*	*BLI and DUT inhibition *^*#*^	In this work

*Repressor forms significant amount of dimers in solution at 10 nM repressor concentration.

^#^*Estimated assuming no competition of substrate binding*.

## Conclusion

Oligomerization of repressor proteins allows for precise regulation of gene expression of various pathogens. In case of pathogenicity islands in Staphylococcus species regulatory mechanisms are governed by the formation of repressor:antirepressor complexes. Several staphylococcal pathogenicity islands employ repressors to regulate their life cycle, and phages interfere with this regulation by distinct antirepressor proteins that are encoded in the phage genomes. Such repressors are frequently dimers on their own and their dimerization is disrupted by the antirepressors. Dimerization characteristics are therefore highly important but yet unexplored due to the exceedingly strong interaction between the repressor subunits, rendering the determination of the relevant dissociation constants hardly measurable in usual assays. To overcome this problem, in the present study, we developed a method exploiting the interactions of two Stl repressor proteins with their antirepressor-mimicking interaction partners allowing the determination of the Stl dimerization constant in the subnanomolar range. This highly efficient, very strong dimerization of the Stl repressors consequently requires an intricate, fine-tuned interaction network between repressor:antirepressor complexation for disruption of the dimer. We also showed for the first time that the strong interaction within the Stl repressor dimer dramatically interferes with the measurement of repressor:antirepressor equilibrium binding constant. Our results thus propose a generally applicable experimental approach to study other similar complexes.

### Supplementary Information

Supplementary Information.

## Data Availability

All data generated or analyzed during this study are included in this published article [and its supplementary information files].
