# Supplementary material for: Antirepressor specificity is shaped by highly efficient dimerization of the staphylococcal pathogenicity island regulating repressors: Stl repressor dimerization perturbed by dUTPases
Source: Sci Rep. 2024 Jan 23;14:1953. doi: 10.1038/s41598-024-51260-y (PMC10806181; doi:10.1038/s41598-024-51260-y)

SUPPORTING INFORMATION

Antirepressor specificity is shaped by highly efficient dimerization of the staphylococcal pathogenicity island regulating repressors: Stl repressor dimerization perturbed by dUTPases

Kinga Nyíri[1,2,*], Enikő Gál[1,2], Máté Laczkovich[1,2], Beáta G. Vértessy[1,2]

[1] Department of Applied Biotechnology and Food Science, Faculty of Chemical Technology and Biotechnology, Budapest University of Technology and Economics, Műegyetem rkp. 3., H-1111 Budapest, Hungary

[2] Institute of Enzymology, Research Centre for Natural Sciences, Hungarian Academy of Sciences, Budapest, Magyar tudósok krt 2. H-1117, Hungary

[*] To whom correspondence should be addressed. Email: nyiri.kinga@vbk.bme.hu

Supplementary Methods

Chemical crosslinking

1% PFA was added to solution of Sa-Stl and Sho-Stl of 1.5 μM concentration. The samples were subjected to SDS gelelectorphoresis (200 V, 45 min) after 30 minutes of incubation at room temperature. Resulting gel was stained with Page Blue protein staining solution.

Size exclusion chromatography

Proteins were subjected to gelfiltration onto a GE Healthcare S200 Increase 10/300 (24 ml) column in a buffer consisting of 50 mM HEPES, pH = 7.5, 300 mM NaCl.

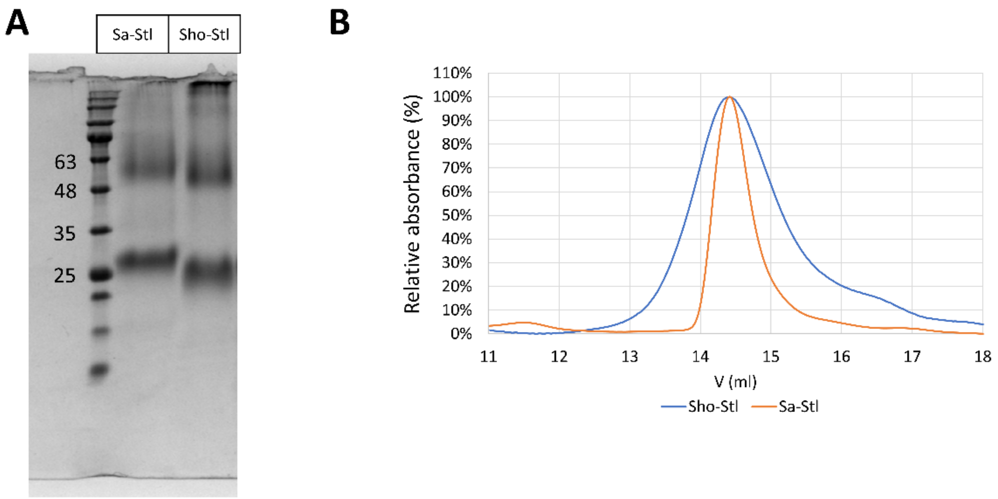

Figure S1. Chemical crosslinking (A) and gelfiltration (B) of Sho-Stl. Both experimental results indicate that the Sho-Stl protein forms dimers as Sa-Stl

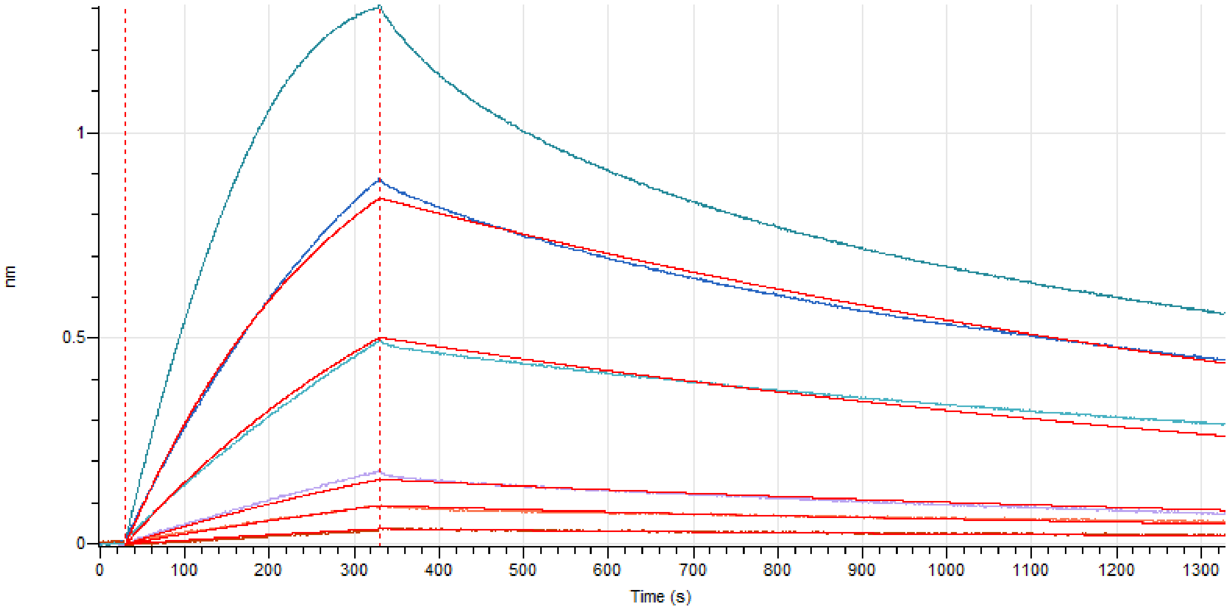

Figure S2. BLI curves of Avi-tagged human dUTPase and Sa-Stl

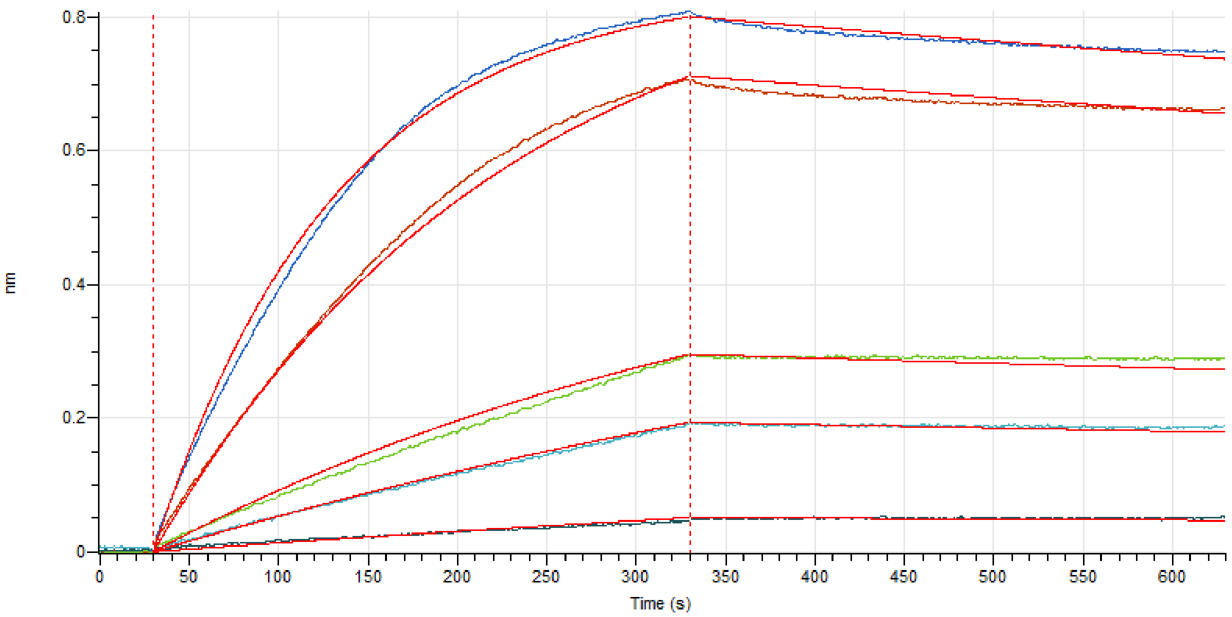

Figure S3. BLI curves of Avi-tagged Sa-Stl and human dUTPase

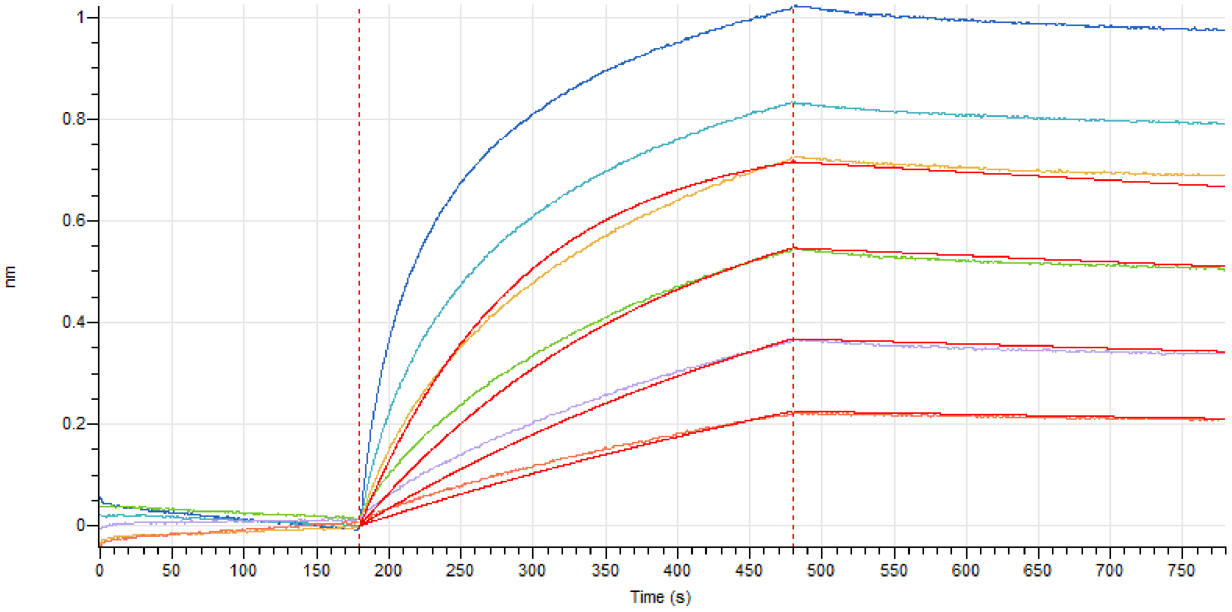

Figure S4. BLI curves of Avi-tagged Sho-Stl and human dUTPase

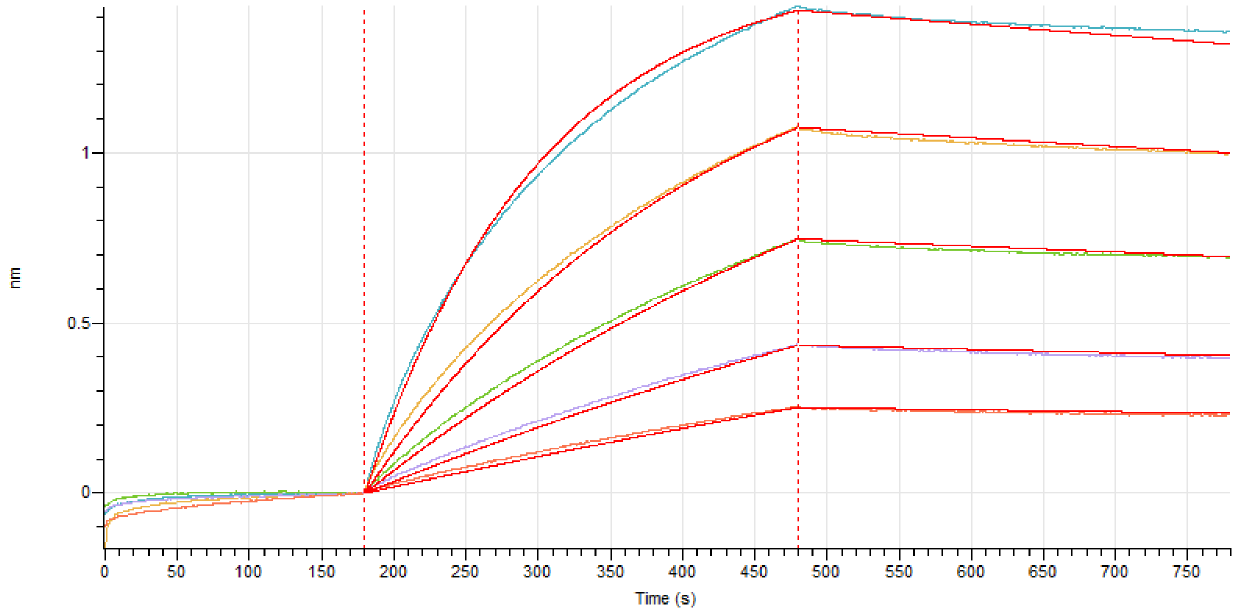

Figure S5. BLI curves of Avi-tagged Sho-Stl-NDY and human dUTPase

hDUT + Sho-Stl (strongly distorted binding curves)

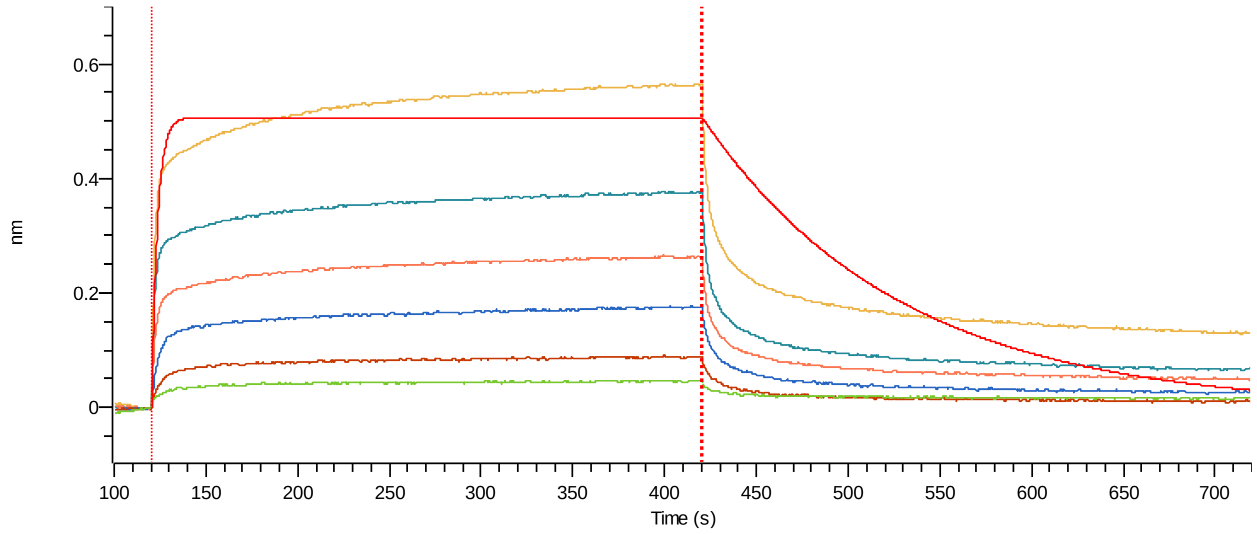

Figure S6. BLI curves of Avi-tagged human dUTPase and Sho-Stl Strongly distorted association curves, with a shape resembling for mass transport limitation in the system, the dissociation fast as that is facilitated by the dimer formation.

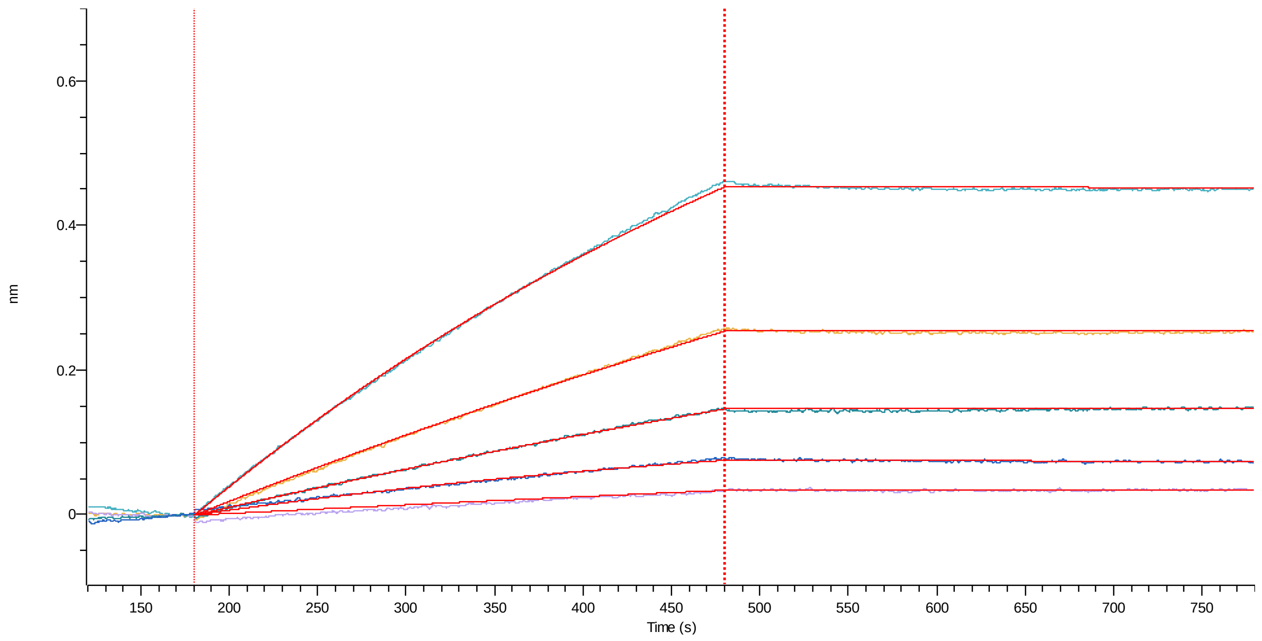

Figure S7. BLI curves of Avi-tagged Sho-Stl and mycobacterial dUTPase

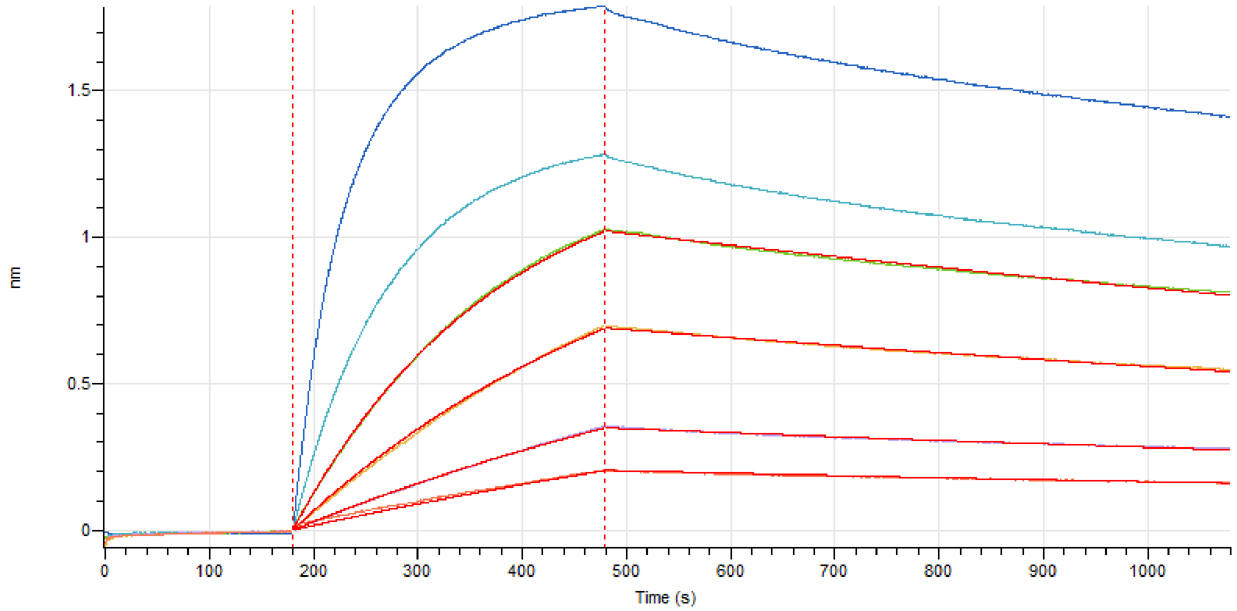

Figure S8. BLI curves of Avi-tagged mycobacterial dUTPase and Sho-Stl

Table S1. List of mutagenesis and cloning primers

Name	sequence (5’→3’)
hDUT-avi-F	TATT**CTCGAG**T<u>ATGGGCAGCAGCC</u>
hDUT-avi-R	GTCC**GGTACC**<u>TTAATTCTTTCCAGTGGAAC</u>
SaStl-avi-F	GGCTCAGAAAATCGAATGGCACGAATAACTCGAGCGGCCGCAT
SaStl-avi-R	TCGAAGATGTCGTTCAGGCCGGACATGTTGGTATCTTTTTCCAGAATAATTTTTTTCTGATGTTC
ShoStl-avi-F	TCAGAAAATCGAATGGCACGAATAAGCGGCCGCATCGTGA
ShoStl-avi-R	GCCTCGAAGATGTCGTTCAGGCCCTTGCTGTTTTTAATCTGTTCAATGAATTCATCC
Sho-Stl-ND-F*	TTATCCACGAAGTTATAGATAC
Sho-Stl-ND-R	AGCCTATTATAACGATGGTGATATCTACTTC
Sho-Stl-Y-F*	TGATATCTACTACAGCAGCTATG
Sho-Stl-Y-R	CCATCGTTATAATAGGCTTTATC
ShoStl-NDY-avi-F	TCAGAAAATCGAATGGCACGAATAAGCGGCCGCATCGTGA
ShoStl-NDY-avi-R	GCCTCGAAGATGTCGTTCAGGCCCTTGCTGTTTTTAATCTGTTCAATGAATTCATCC
mtDUT-avi-F	TATGCTCGAGTATGGGCAGCAGCCATCATC
mtDUT-avi-R	GCAGGTACCTCACAAACTCGCATGTCCG

* Sho-Stl-ND-F/R and Sho-Stl-Y-F/R primers were applied in 2 subsequent rounds of mutagenesis to result in the triple mutant Sho-Stl-NDY

SDS PAGE images of the final protein preparations used in this study

Sa-Stl

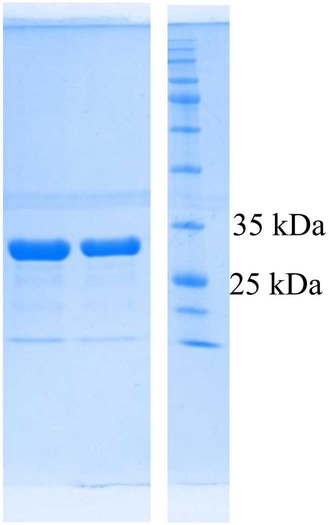

Sa-Stl-avi

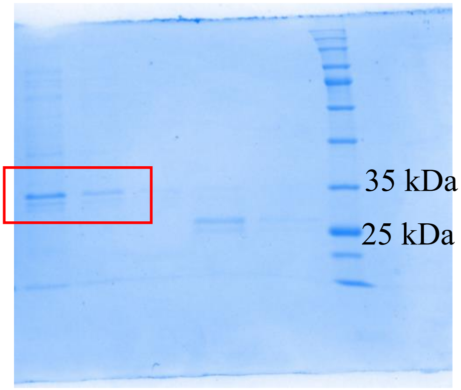

Sho-Stl

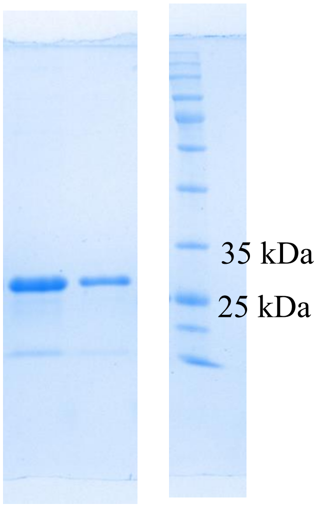

Sho-Stl-avi

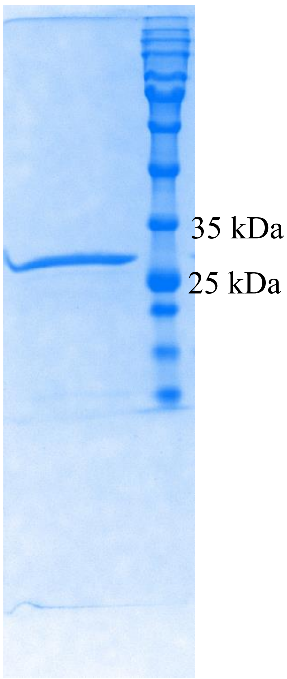

Sho-Stl-NDY

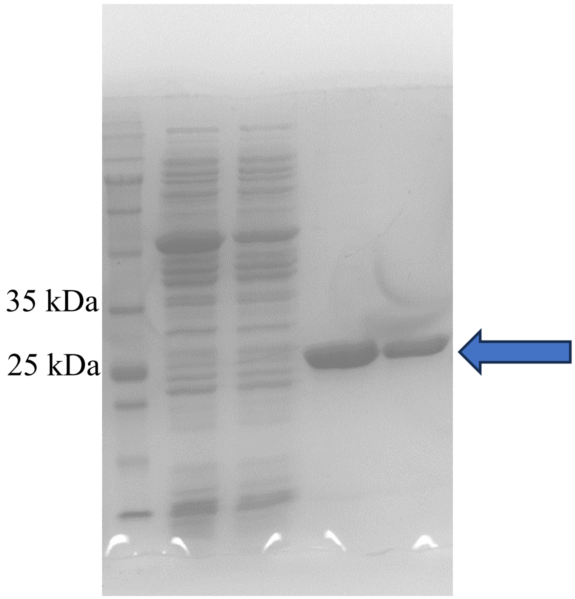

Sho-Stl-NDY-avi

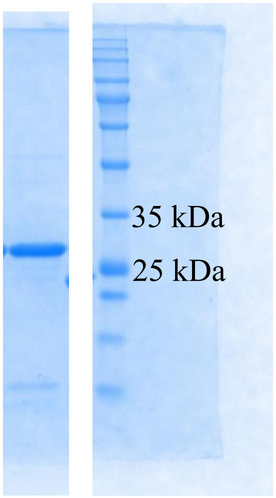

hDUT

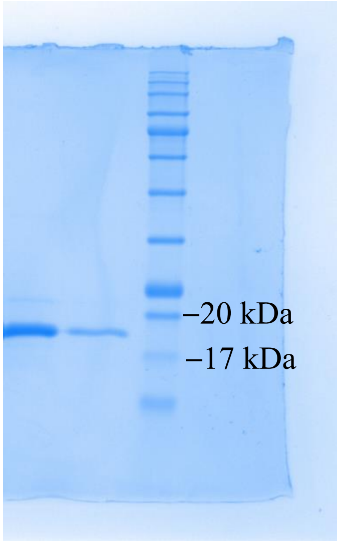

hDUT-avi

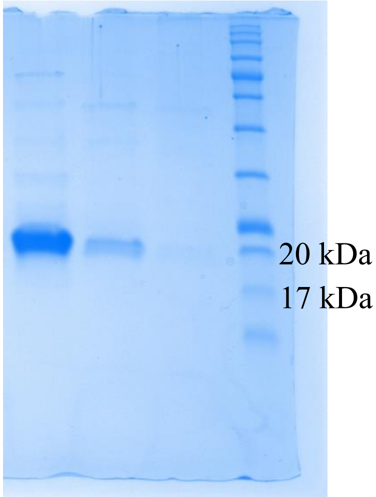

MtDUT-avi

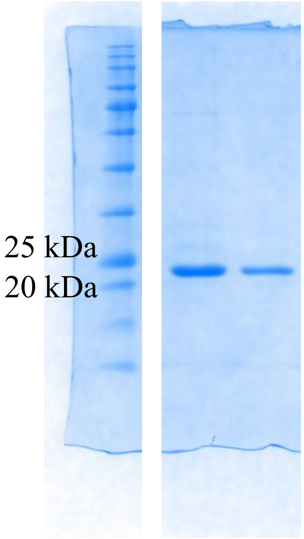

MtDUT

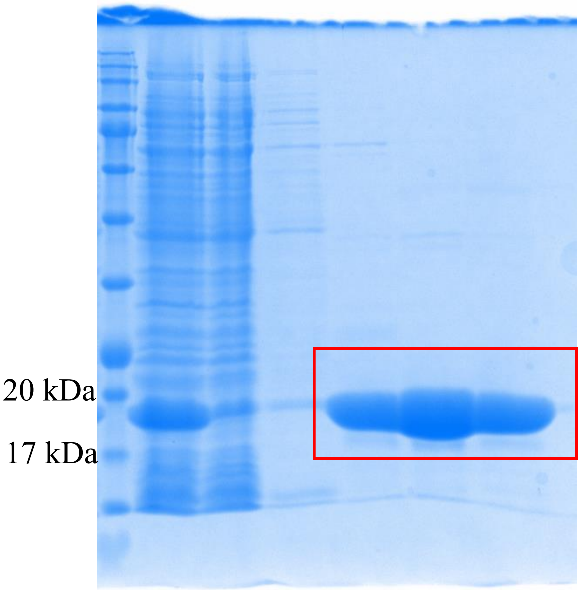

Supplement: Supplementary file 1 — Supplementary Information. [file 41598_2024_51260_MOESM1_ESM.pdf]
